# Supplementary material for: Phenylalanine Increases the Production of Antioxidant Phenolic Acids in Ginkgo biloba Cell Cultures
Source: Molecules. 2021 Aug 17;26(16):4965. doi: 10.3390/molecules26164965 (PMC8400538; doi:10.3390/molecules26164965)
Supplement: Supplementary file 1 [file molecules-26-04965-s001.zip › molecules-1323042-supplementary.pdf]

Supplementary files

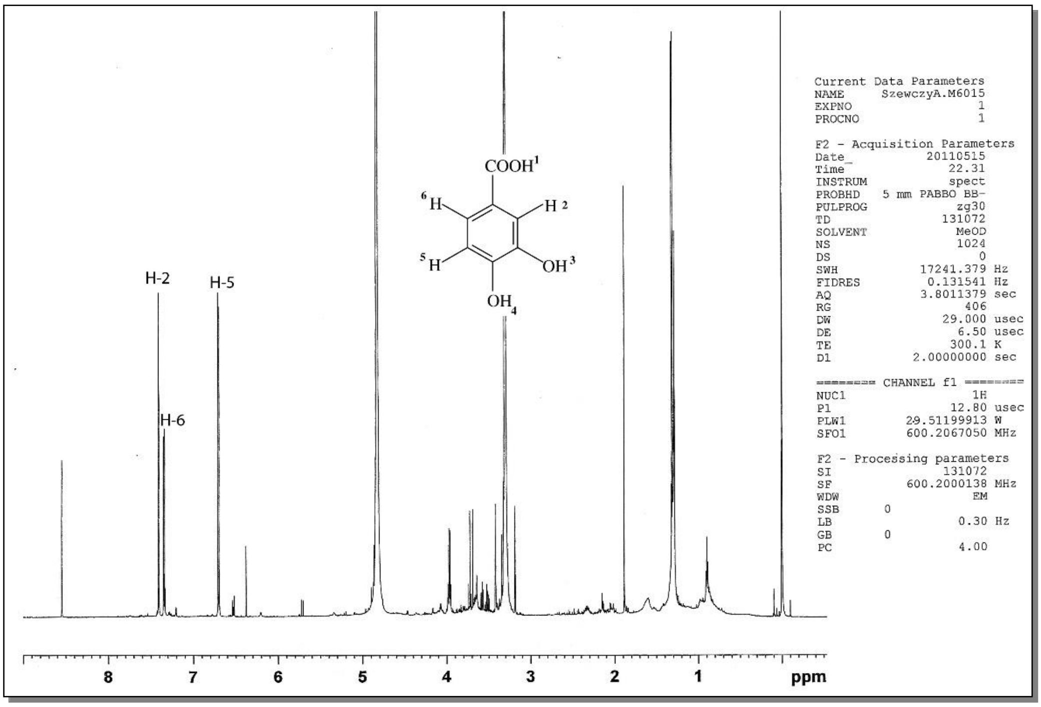

Figure S1 ^{1}HNMR spectrum of compound 1 (protocatechuic acid)

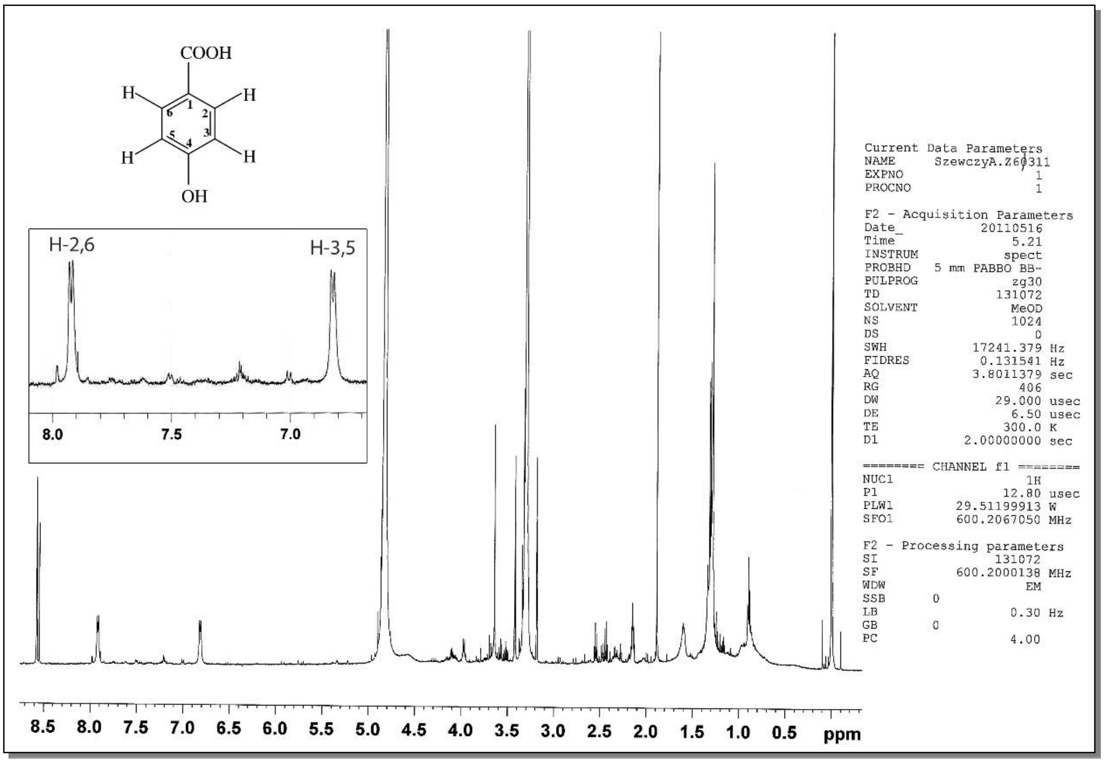

Figure S2 ^{1}HNMR spectrum of compound 2 (*p*-hydroxybenzoic acid)

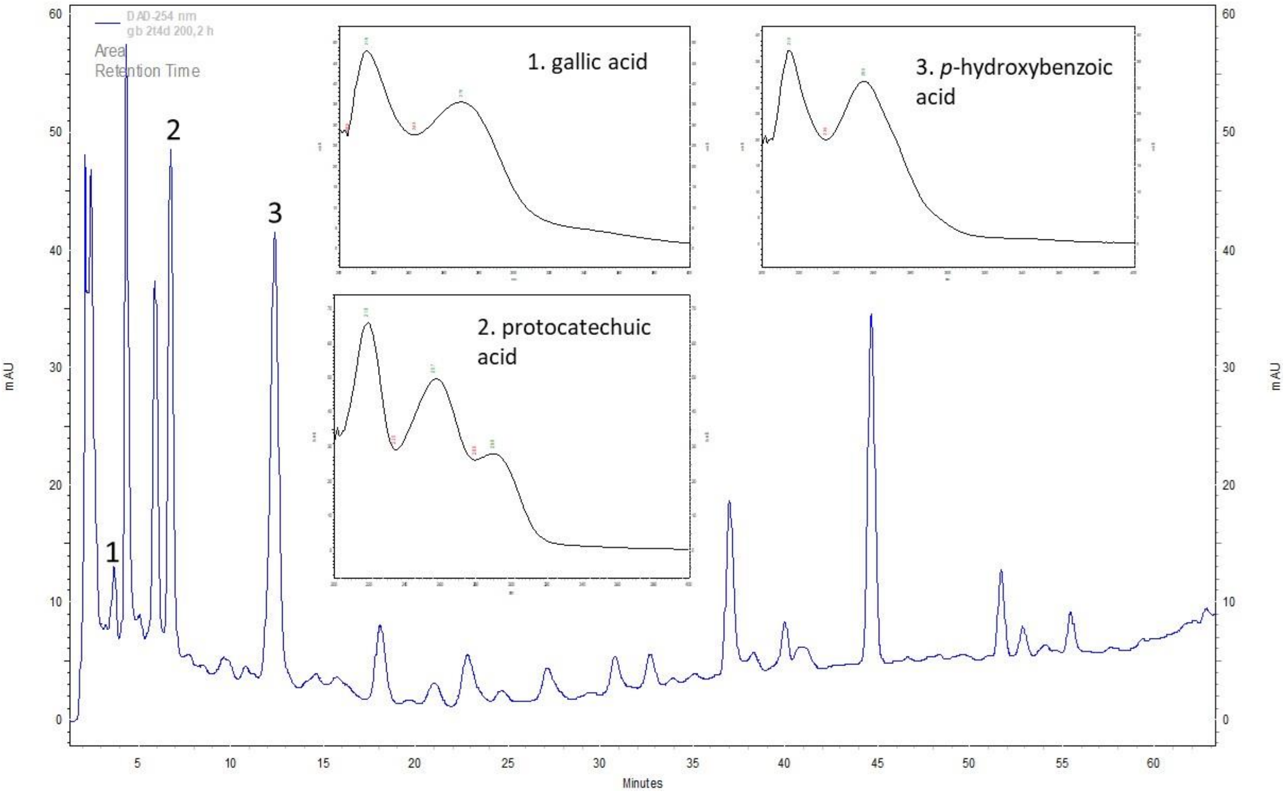

Figure S3 Sample chromatogram and UV spectra of the extract from Ginkgo biloba cell cultures (4 days after addition of 200 mg of phenylalanine, week 2).

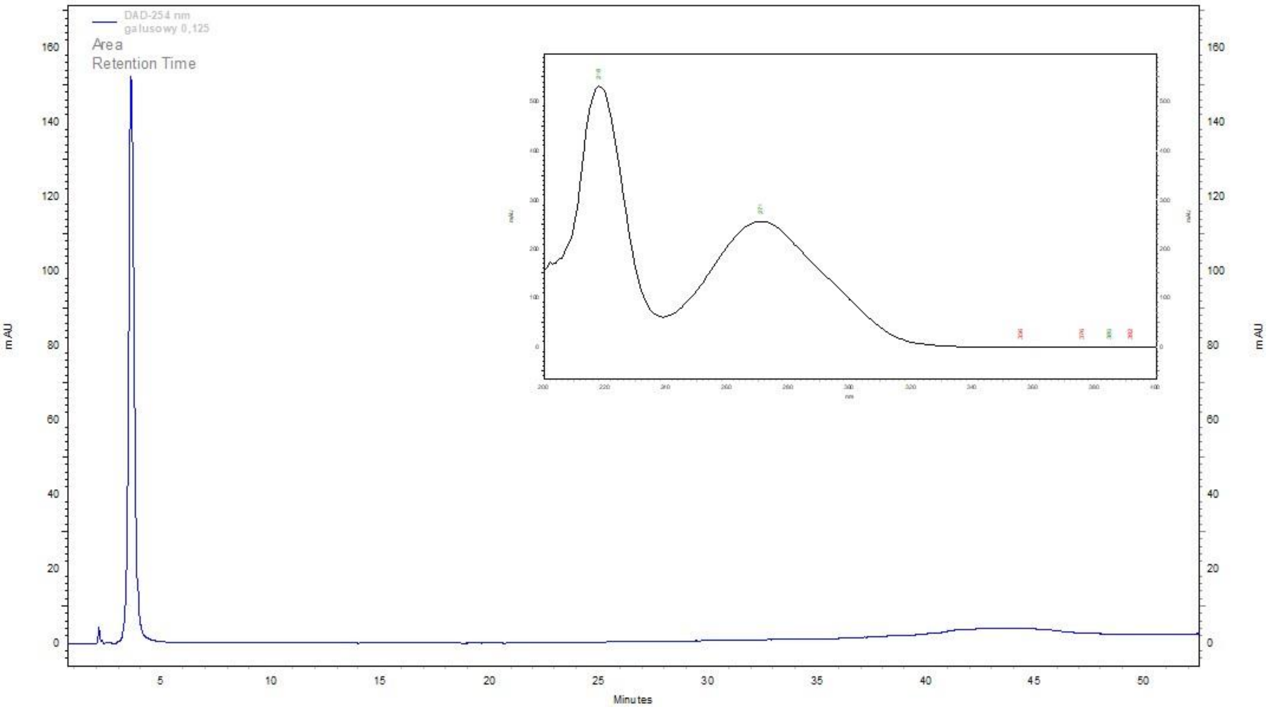

Figure S4 Chromatogram and UV spectrum of gallic acid.

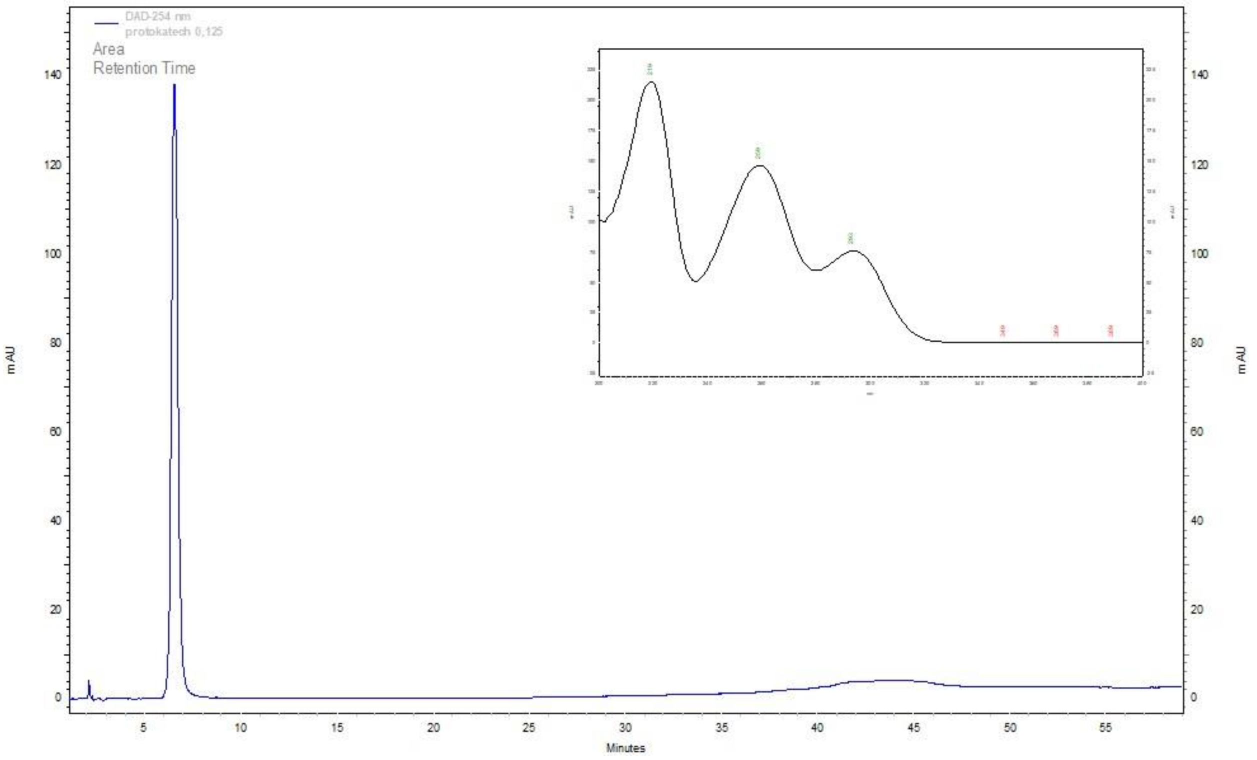

Figure S5 Chromatogram and UV spectrum of protocatechuic acid.

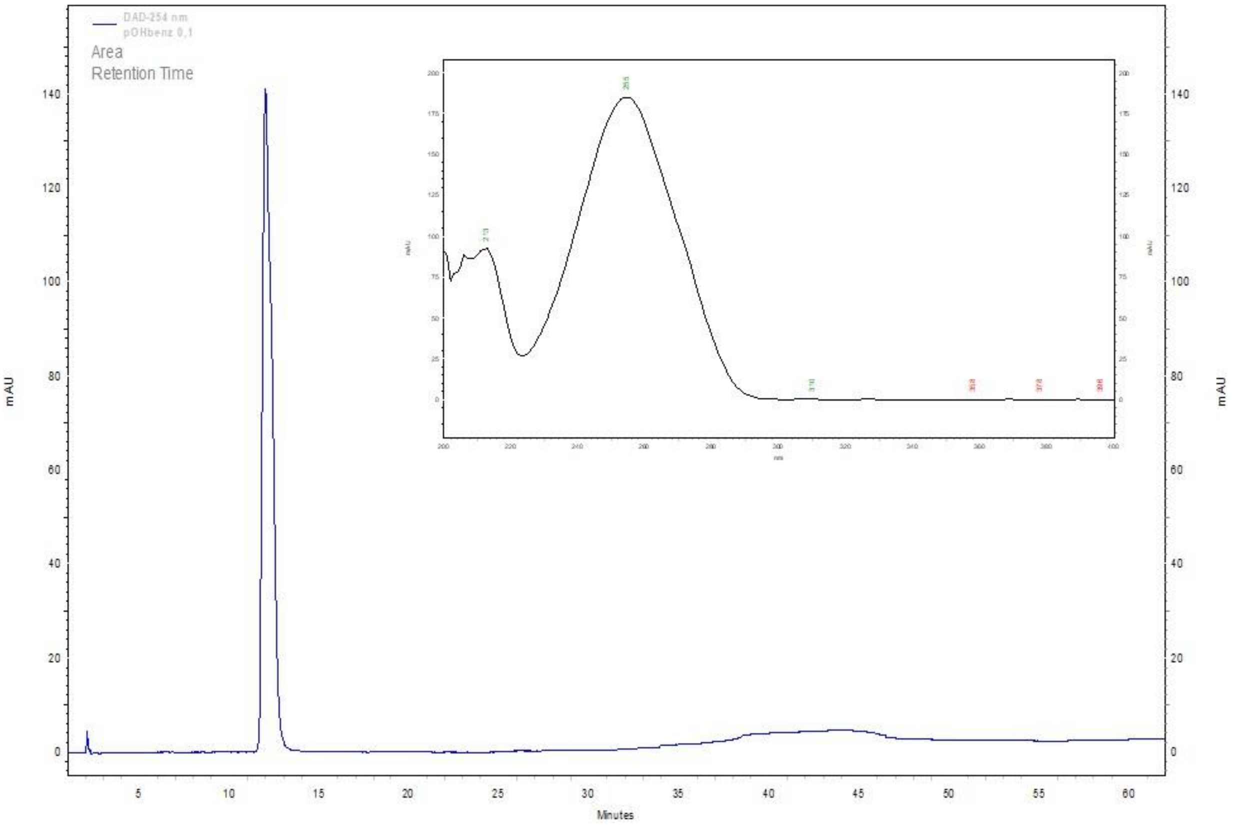

Figure S6 Chromatogram and UV spectrum of *p*-hydroxybenzoic acid.

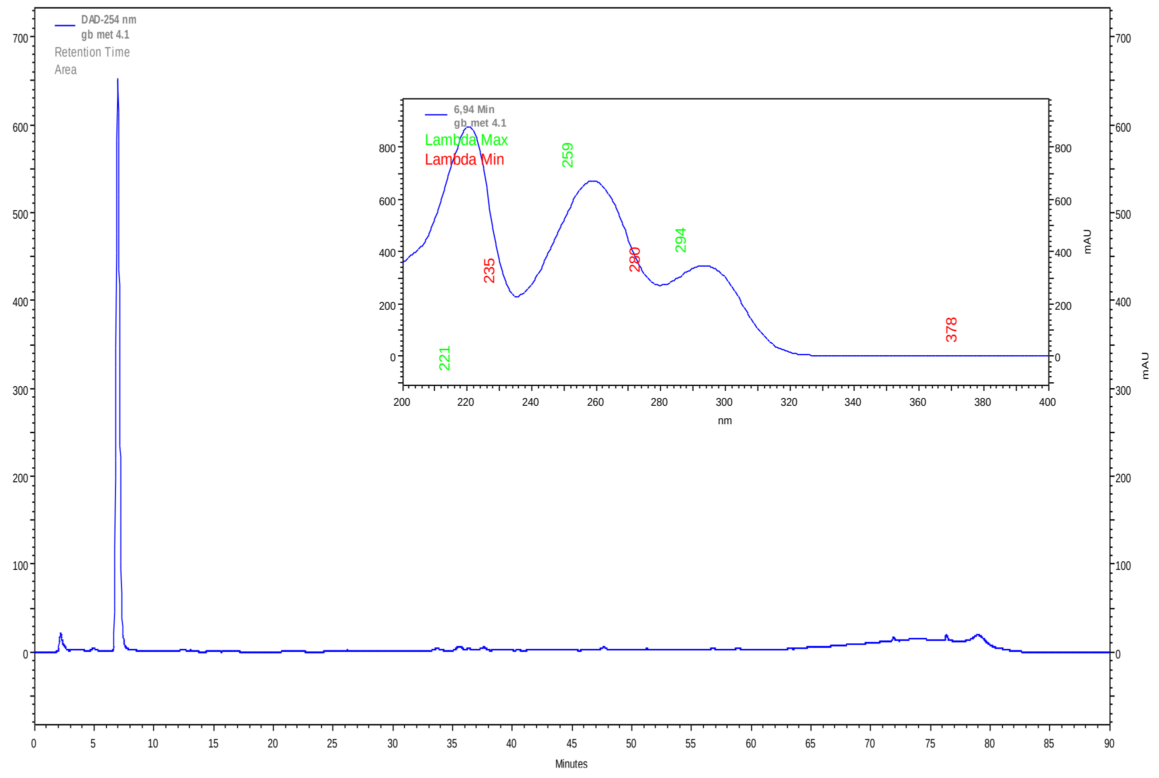

Figure S7 Chromatogram and UV spectrum of compound 1 (protocatechuic acid)

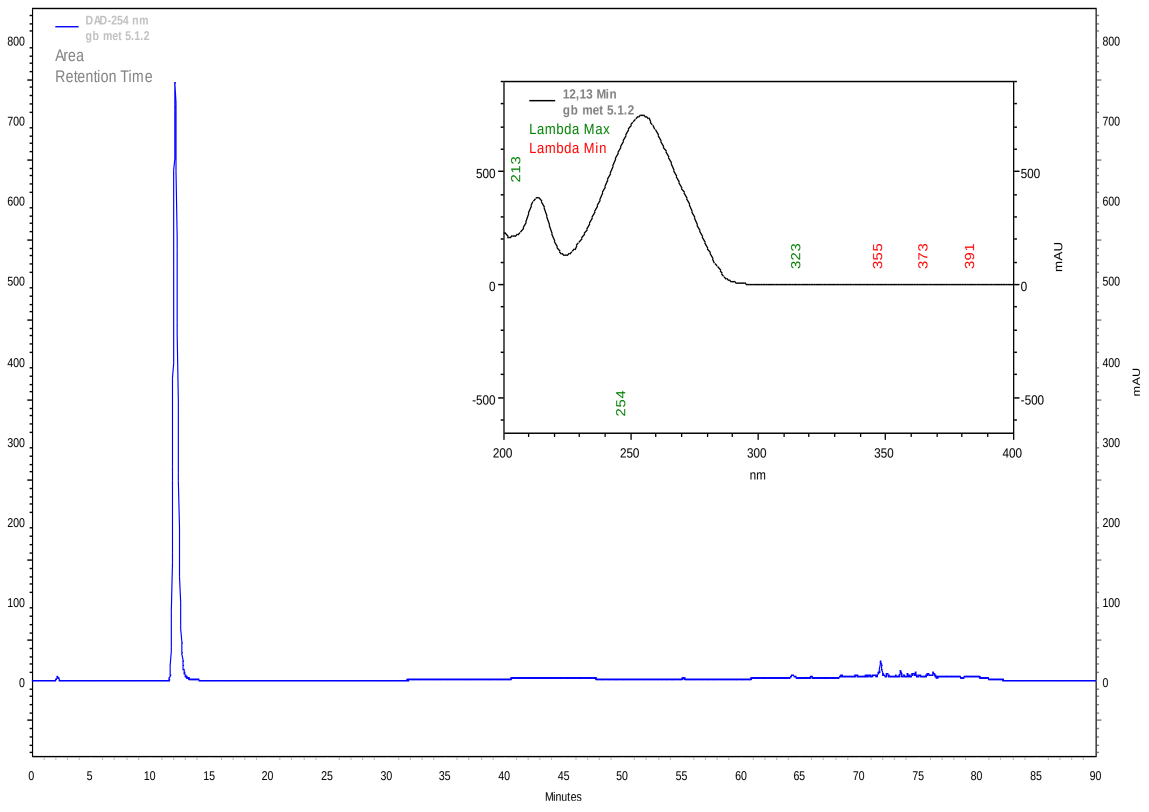

Figure S8 Chromatogram and UV spectrum of compound 2 (*p*-hydroxybenzoic acid)
